# Discovery of cell-type-specific mtDNA common deletion in nasopharyngeal carcinoma​

**DOI:** 10.1093/bib/bbaf631.057

**Published:** 2025-12-12

**Authors:** Mun Kay Ho, Joshua W K Ho

**Affiliations:** School of Biomedical Sciences, Li Ka Shing Faculty of Medicine, The University of Hong Kong, Hong Kong SAR, China; School of Biomedical Sciences, Li Ka Shing Faculty of Medicine, The University of Hong Kong, Hong Kong SAR, China

## Abstract

**Background:**

The human mitochondrial genome (mtDNA) plays a key role in maintaining cellular functions. However, mtDNA is vulnerable to mutations due to the lack of protective histones and low efficiency in DNA repair mechanisms. Among these mutations, the 4977 bp mtDNA common deletion (mtDNA-CD) encompasses twelve essential genes that are essential for oxidative phosphorylation.

**Aim:**

In previous studies, mtDNA-CD was identified in nasopharyngeal carcinoma (NPC) patient samples. However, bulk sequencing protocols employed in past studies were unable to distinguish whether those mtDNA-CD were specific to the cancer cells or from other non-cancer cell types in the samples. Single-cell RNA sequencing (scRNA-seq) enables the discovery of different cell types within a population and facilitates the identification of cell-type-specific mutation signatures. In this study, we propose to use scRNA-seq data to identify cell-type-specific deletion signatures in NPC samples.

**Methods:**

We developed a custom computational pipeline to detect large-scale deletions using scRNA-seq data. The pipeline harnesses both clipped read profiles and secondary alignments to overcome the typical limitations of low sequencing depth and uneven read coverage in scRNA-seq data. We tested our pipeline on an NPC data set consisting of nine patients.

**Results:**

Our result suggests the presence of mtDNA-CD mutations specific to malignant cancer cells in NPC tumour samples. This study proposes the use of scRNA-seq of cell-type-specific mtDNA-CD in NPC samples, offering new insights into the genetic and molecular pathogenesis of cancer and guiding the development of specific biomarkers for NPC.

